# Add-on therapy in metformin-treated patients with type 2 diabetes at moderate cardiovascular risk: a nationwide study

**DOI:** 10.1186/s12933-020-01078-5

**Published:** 2020-07-06

**Authors:** David Thein, Mia Nielsen Christiansen, Ulrik Madvig Mogensen, Johan Skov Bundgaard, Rasmus Rørth, Christian Madelaire, Emil Loldrup Fosbøl, Morten Schou, Christian Torp-Pedersen, Gunnar Gislason, Lars Køber, Søren Lund Kristensen

**Affiliations:** 1 Department of Cardiology, Rigshospitalet, Copenhagen University Hospital, Rigshospitalet Inge Lehmanns vej 7, 2100 Copenhagen Ø, Denmark; 2 Department of Cardiology, Herlev and Gentofte Hospital, Copenhagen University Hospital, Copenhagen, Denmark; 3 Department of Health, Science and Technology, Aalborg University, Aalborg, Denmark; 4 The Danish Heart Foundation, Copenhagen, Denmark; 5 The National Institute of Public Health, University of Southern Denmark, Copenhagen, Denmark

**Keywords:** Type 2 diabetes, Heart failure, Myocardial infarction, Prognosis, Treatment

## Abstract

**Background:**

In randomised clinical trials, glucagon-like peptide-1 receptor agonists (GLP-1 RAs) and sodium–glucose cotransporter 2 (SGLT-2) inhibitors reduced cardiovascular events in patients with type 2 diabetes (T2D) at high cardiovascular risk, as compared to standard care. However, data comparing these agents in patients with T2D who are at moderate risk is sparse.

**Methods:**

From Danish national registries, we included patients with T2D previously on metformin monotherapy, who started an additional glucose-lowering agent [GLP-1 RA, SGLT-2 inhibitor, dipeptidyl peptidase-4 (DPP-4) inhibitor, sulfonylurea (SU), or insulin] in the period 2010-2016. Patients with a history of cardiovascular events [heart failure (HF), myocardial infarction (MI) or stroke] were excluded. Patients were followed for up to 2 years. Cause-specific adjusted Cox regression models were used to compare the risk of hospitalisation for HF, a composite endpoint of major adverse cardiovascular events (MACE) (MI, stroke or cardiovascular death), and all-cause mortality for each add-on therapy. Patients who initiated DPP-4 inhibitors were used as reference.

**Results:**

The study included 46,986 T2D patients with a median age of 61 years and of which 59% were male. The median duration of metformin monotherapy prior to study inclusion was 5.3 years. Add-on therapy was distributed as follows: 13,148 (28%) GLP-1 RAs, 2343 (5%) SGLT-2 inhibitors, 15,426 (33%) DPP-4 inhibitors, 8917 (19%) SUs, and 7152 (15%) insulin. During follow-up, 623 (1.3%, range 0.8-2.1%) patients were hospitalised for HF—hazard ratios (HR) were 1.11 (95% CI 0.89–1.39) for GLP-1 RA, 0.84 (0.52–1.36) for SGLT-2 inhibitors, 0.98 (0.77–1.26) for SU and 1.54 (1.25–1.91) for insulin. The composite MACE endpoint occurred in 1196 (2.5%, range 1.5–3.6%) patients, yielding HRs of 0.82 (0.69–0.97) for GLP-1 RAs, 0.79 (0.56–1.12) for SGLT-2 inhibitors, 1.22 (1.03–1.49) for SU and 1.23 (1.07–1.47) for insulin. 1865 (3.9%, range 1.9–9.0%) died from any cause during follow-up. HRs for all-cause mortality were 0.91 (0.78–1.05) for GLP-1 RAs, 0.79 (0.58–1.07) for SGLT-2 inhibitors, 1.13 (0.99–1.31) for SU and 2.33 (2.08–2.61) for insulin.

**Conclusion:**

In a nationwide cohort of metformin-treated T2D patients and no history of cardiovascular events, the addition of either GLP-1 RA or SGLT-2 inhibitor to metformin treatment was associated with a similar risk of hospitalisation for HF and death, and a lower risk of MACE for GLP-1 RA when compared with add-on DPP-4 inhibitors. By contrast, initiation of treatment with SU and insulin were associated with a higher risk of MACE. Additionally, insulin was associated with an increased risk of all-cause mortality and hospitalisation for HF.

## Background

In patients with type 2 diabetes (T2D), cardiovascular disease is the primary cause of death, often due to an increased risk of myocardial infarction (MI), stroke, and heart failure (HF) [1–4]. The prevalence of HF is > 20% in patients with T2D aged ≥ 65 years. This group of patients is at an increased risk of death, with an expected median time lifetime of 4–5 years for combined HF, T2D and age ≥ 65 years [5].

In recent guidelines concerning T2D, the recommendations on first- and second-line therapy have been updated, and take into account the cardiovascular (CV) risk profile of the patient [6–8]. For patients considered to have atherosclerotic CV disease or be at a high or very high CV risk, Glucagon-like peptide-1 receptor agonists (GLP-1 RA) or sodium–glucose cotransporter 2 (SGLT-2) inhibitors are recommended over dipeptidyl peptidase-4 (DPP-4) inhibitors or sulfonylurea (SU) as add-on therapy to metformin—and in certain cases, GLP-1 RAs and SGLT-2 inhibitors are recommended as first line therapy. These recommendations are based on CV outcome trials that included patients with T2D and either an established CV disease or a high CV risk profile [6–8]. These trials have shown a reduction in atherosclerotic events with GLP-1 RAs, and for SGLT-2 inhibitors a reduction in hospitalisation for HF, adverse renal outcomes and all-cause mortality [9–18]. Cardiovascular outcome trials on dipeptidyl peptidase-4 (DPP-4) inhibitors have not demonstrated similar cardiovascular or renal benefits [19–23].

Importantly, meta-analyses have suggested that the cardiovascular benefits of GLP-1 RA and SGLT-2 inhibitors may primarily be present in patients where treatment is administered as a secondary prevention initiative e.g. in patients with an established CV disease [17, 24]. Previous observational studies have shown SGLT-2 inhibitors to be associated with improved cardiovascular outcomes, but in these studies the majority of patients had established CV disease [12, 16].

The aim of the present study is to investigate whether the benefits associated with use of GLP-1 RAs and SGLT-2 inhibitors extend to patients with T2D at a lower CV risk in a nationwide Danish cohort. Thus, we compare the incidence of cardiovascular events in relation to add-on glucose-lowering therapy in patients who initiated second-line add-on therapy to metformin and had no history of CV events [16, 25].

## Methods

### Setting

The Danish health-care system is based on the Beveridge model, offering free access to health services throughout the primary, secondary, and tertiary sector. The prevalence of T2D in Denmark is estimated to be 6%, which is comparable to that of the USA (7%) and the UK (5%) [26, 27]. From 2010 to 2016, the Danish and international guidelines on the treatment of T2D recommended that the treating physician add any of the listed therapies (GLP-1 RA, SGLT-2 and DPP-4 inhibitor, SU, and insulin) alongside metformin, if glycaemic control was not achieved with metformin monotherapy [28].

### Data sources

All Danish residents are assigned a unique personal identification number at birth or upon immigration. This identification allows for linkage of data across different national registries. In this study, we combined data from the following data sources; (1) *The Danish National Patient Registry* which holds information on all hospital admissions since 1978, and outpatient visits since 1995. Diagnoses are coded according to the International Classification of Diseases (ICD-10). The ICD-10 codes used for outcomes in the present study have been validated and have a positive predictive value of > 90% for the outcomes of MI, stroke, and HF [29, 30]. (2) *The Danish Register of Medicinal Product Statistics* (also known as the national prescription registry) contains information on all dispensed prescriptions since 1995. The international Anatomical Therapeutical Chemical (ATC) system is used to classify dispensed drugs [31]. National Pharmacies are required by law to register all dispensed prescriptions due to the national subsidiaries on drug expenses. (3) *The National Population Registry* contains information on sex, vital status, date of birth, and, if applicable, date of death.

### Study population and baseline variables

**Fig. 1:**
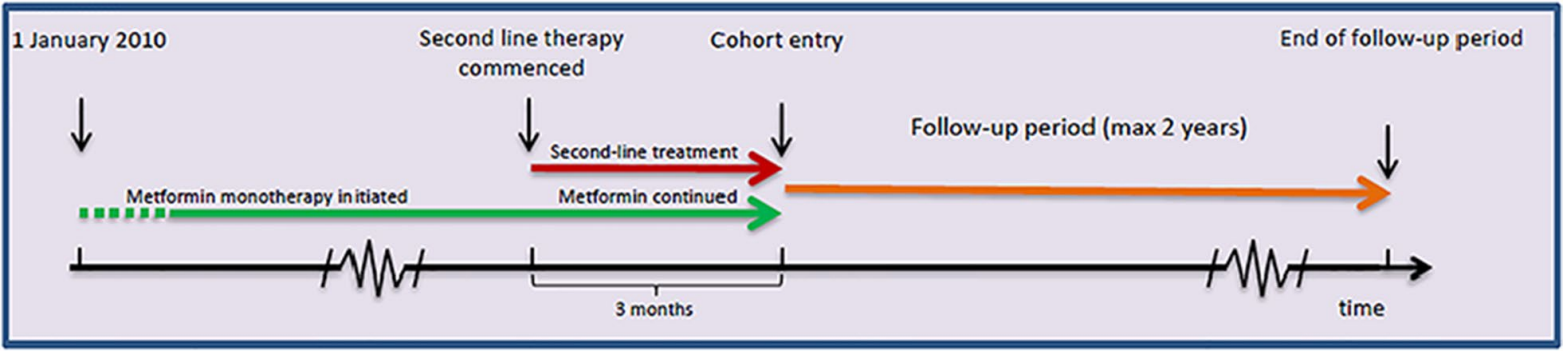
Graphical representation of the criteria for add-on therapy

The study population was composed of patients with T2D on metformin monotherapy who initiated add-on therapy between the 1st of January 2010 and the 31st of December 2016. Patients with T2D were defined as those with the presence of ICD-10 code E11 from the Danish National Patient Registry or a filled prescription for metformin. Initiation of second-line add-on therapy (GLP-1 RA, SGLT-2 inhibitor, DPP-4 inhibitor, SU, or insulin) was defined by the following criteria; (1) a filled prescription for one of the examined glucose-lowering therapies (2) no previous history of any glucose-lowering therapy apart from metformin, and (3) a filled prescription for metformin during a 6 month period prior to the beginning of the add-on therapy, and again during a 3 month period after the initiation of add-on therapy (Fig. 1). These criteria were applied to ensure that the included patients required intensified treatment for T2D. Consequently, the date of inclusion was set 3 months after the initiation of add-on therapy to avoid immortal time bias. Patients were excluded if they had a history of hospitalisation for HF, MI, or stroke prior to the date of inclusion.

Comorbidities were defined by the presence of relevant ICD-10 codes acquired during hospitalisation over a 10-year period leading up to the date of inclusion. The comorbidities included were hypertension, atrial fibrillation, cancer, chronic obstructive pulmonary disease, ischemic heart disease, peripheral atherosclerosis, renal disease, and thyroid disease (for ICD-10 codes see Additional file 1: Table S1). Ongoing pharmacotherapy was defined by at least one filled prescription for any of the following drugs during the 6 months prior to the date of inclusion: Angiotensin-converting enzyme (ACE) inhibitors, angiotensin-II blockers inhibitors, acetylsalicylic acid, calcium channel blockers, digoxin, clopidogrel, mineralocorticoid receptor antagonists, statins, β-blockers and loop diuretics (for ATC codes see Additional file 1: Table S1). The duration of metformin monotherapy was estimated as the time difference between the first filled prescription for metformin and the initiation of the respective add-on therapy. The CV risk of the included patients was assessed in accordance with the recently published EASD/ESC guidelines on management of CV risk in diabetes within the limits of the national patient registries [6]. Patients were classified as being at moderate risk due to their T2D status alone. Additionally, patients were deemed to be at high risk if the duration of T2D exceed 10 years, and at very high risk if the patient had a history of peripheral vascular disease, ischemic heart disease, or chronic kidney disease.

### Stratification, exposure, and outcomes

Patients were grouped according to the initiated add-on therapy (GLP-1 RAs, SGLT-2 inhibitors, DPP-4 inhibitors, SU, or insulin). Patients were followed until an event occurred, the 1st of August 2017, or for a maximum follow-up of 2 years. The two-year follow-up was chosen based on a preliminary analysis on the median time patients remained in the same add-on therapy group. For analyses, patients stayed in their initial assigned group irrespective of potential changes in treatment throughout follow-up. This approach was chosen to avoid unnecessary complexity in the interpretation of results, as changes in glucose-lowering treatment during follow-up may be influenced by a multitude of factors including possible suspicion of cardiovascular disease. The primary outcome of the study was hospitalisation for HF. The study had two secondary outcomes; a composite endpoint of major adverse cardiovascular events (MACE) in the form of MI, stroke or CV death, and all-cause mortality. Hospitalisation for HF was chosen as the primary endpoint due to the results presented in the CV outcome trials indicating beneficial effects on this outcome. Discontinuation of treatment or introduction of insulin was assessed in sensitivity analyses. The group starting DPP-4 inhibitor treatment was chosen as reference, as it included the majority of patients and underwent an initial introduction to market in the studied time period, which was also the case for GLP-1 RA and SGLT-2 inhibitors.

### Statistics

For baseline characteristics, differences between groups were compared by ANOVA or Friedman test for continuous variables, and Pearson’s chi-squared test for categorical variables. P-values were reported for ANOVA and Friedman test. Categorical variables were compared with DPP-4 inhibitors (reference) by Bonferroni corrected multiple comparison and statistically significant differences were highlighted. The primary outcome of hospitalisation for HF and the secondary outcomes of MACE and all-cause mortality were analysed by cause-specific Cox-proportional hazard regression adjusted for age, sex, comorbidities (hypertension, atrial fibrillation, cancer, chronic obstructive pulmonary disease, ischemic heart disease, peripheral atherosclerosis, renal disease, microvascular complications, and thyroid disease), use of statins, CV risk profile, duration of metformin monotherapy, and year of inclusion. DPP-4 inhibitors were used as reference. We tested for interaction between treatment effects, sex, and age respectively by a likelihood ratio test and found no significant interactions unless stated otherwise. All parameters were tested to be in accordance with the proportional hazard assumptions. Event rates were calculated per 1000 person-years, accounting for the competing risk of death. For all analyses, a *p* value < 0.05 was considered statistically significant. The following five sensitivity analyses were conducted: (1) Patients were followed until a prescription was filled for any anti-diabetic therapy different from the initial treatment, (2) Follow-up was extended to 3 years, (3) Patients were stratified according to their CV risk (4) The cohort was split in two based the date of inclusion (before and after September 2013), (5) SU was used as reference. All statistical analyses were conducted in the SAS statistical software package, version 9.4 (SAS Institute, Cary, NC).

## Results

### Baseline characteristics

**Fig. 2:**
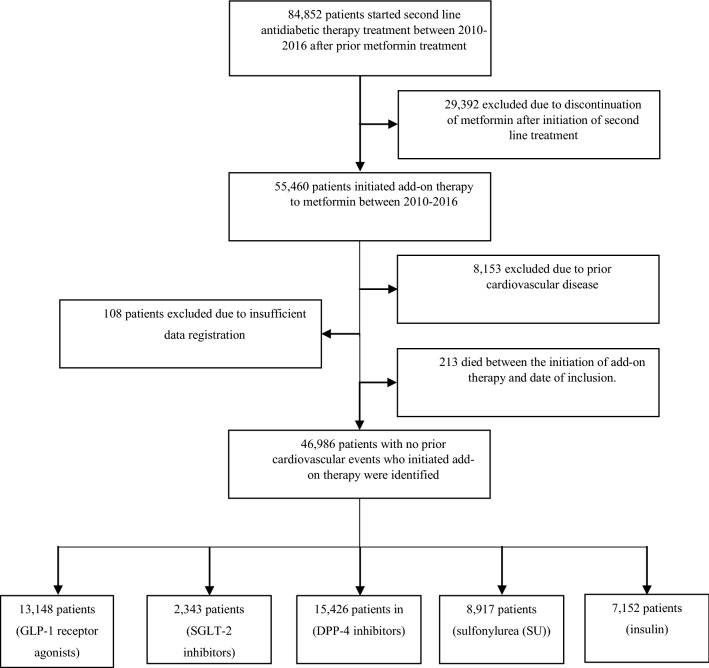
Consort Diagram for the inclusion and exclusion of patients in the study—*GLP-1* glucagon like peptide-1 analogues, *SGLT-2* sodium–glucose transporter 2 inhibitor, *DPP-4* dipeptidyl peptidase inhibitor. Add-on therapy is the introduction of a new glucose-lowering drug in addition to on-going metformin treatment

A total of 55,460 patients on metformin monotherapy fulfilled the inclusion criteria of starting an additional glucose-lowering agent. Of these, 8153 patients were excluded due to a history of MI, stroke, or hospitalisation for HF, 213 were excluded due to the occurrence of death between the initiation of add-on therapy and the date of inclusion, and 108 were excluded due to insufficient data (Fig. 2). The remaining 46,986 patients had the following distribution: 13,148 (28%) in GLP-1 RAs, 2343 (5%) in SGLT-2 inhibitors, 15,426 (33%) in DPP-4 inhibitors, 8917 (19%) in SU, and 7152 (15%) in insulin (Fig. 2). The median duration of metformin monotherapy prior to study inclusion varied from 3 to 7 years across groups. The lowest duration was among patients in SU add-on therapy (3.0 years) and the highest in GLP-1 RAs (6.5 years) and SGLT-2 inhibitors (7.2 years). A majority of patients were men (ranging from 56% among GLP-1 RA and up to 63% in the insulin group), and mean age ranged from 58 to 62 years—GLP-1 RA and DPP-4 inhibitor respectively (Table 1). The number of patients at high or very high cardiovascular risk according to the EASD/ESC criteria was greater among those who started add-on GLP-1 RA or SGLT-2 inhibitor treatment (36% at high or very high risk) as compared to insulin (30%), DPP-4 inhibitors (26%) and SUs (17%). Hypertension was present in 35–50% of patients, less frequently among add-on SU and most frequently in GLP-1 RA. Ischemic heart disease was prevalent in 11–16% of patients, lowest in SU and SGLT-2 inhibitor groups and highest among those in add-on GLP-1 RA. The burden of comorbidities including cancer and renal disease was largest in the insulin group. The majority of patients were treated with statins, ranging from 63% in the SGLT-2 group to 89% in the GLP-1 RA group. Use of ACE inhibitors or angiotensin II receptor blockers were frequently used and ranged from 55% to 69% respectively (Table 1).

**Table 1:** Baseline characteristics according to second-line therapy in addition to metformin

	DPP-4 inhibitors	GLP-1 receptor agonists	SGLT-2 inhibitors	Sulfonylurea	Insulin	p value
Number of patients	15,426 (33%)	13,148 (28%)	2343 (5%)	8917 (19%)	7152 (15%)	
Male	9160 (59%)	7403 (56%)^b^	1398 (60%)	5473 (61%)	4480 (63%)^b^	
Age	62.4 (± 11.9)	58.0 (± 11.2)	60.2 (± 11.3)	59.6 (± 12.0)	61.2 (± 13.2)	< 0.0001
Years in metformin monotherapy	5.1 (± 4.0)	6.5 (± 4.5)	7.2 (± 5.1)	3.0 (± 3.0)	4.7 (± 4.4)	< 0.0001
Follow-up (years)	1.8 (± 0.4)	1.9 (± 0.3)	1.4 (± 0.6)	1.8 (± 0.4)	1.7 (± 0.5)	< 0.0001
Cardiovascular risk^a^						
Moderate	11,370 (74%)	8423 (64%)^b^	1489 (64%)^b^	7401 (83%)^b^	5007 (70%)^b^	
High	1461 (9%)	2157 (16%)^b^	563 (24%)^b^	233 (3%)^b^	758 (11%)	
Very high	2595 (17%)	2568 (20%)^b^	291 (12%)^b^	1283 (14%)^b^	1387 (19%)^b^	
Medical history						
Chronic obstructive pulmonary disease	945 (6%)	920 (7%)	113 (5%)	568 (6%)	652 (9%)^b^	
Hypertension	6145 (40%)	6523 (50%)^b^	841 (36%)^b^	3162 (35%)^b^	3160 (44%)^b^	
Atrial fibrillation	1409 (9%)	1106 (8%)	121(5%)^b^	757 (8%)	741 (10%)	
Cancer	2125 (14%)	1630 (12%)^b^	232 (10%)^b^	1133 (13%)	1373 (19%)^b^	
Thyroid disease	356 (2%)	386 (3%)	57 (2%)	183 (2%)	194 (3%)	
Renal disease	1245 (8%)	1390 (11%)^b^	109 (5%)^b^	477 (5%)^b^	772 (11%)^b^	
Ischemic heart disease	1993 (13%)	2089 (16%)^b^	249 (11%)^b^	1013 (11%)^b^	1008 (14%)	
Peripheral arterial disease	378 (2%)	384 (3%)	37 (2%)	175 (2%)	290 (4%)	
Pharmacotherapy						
Statin	10,902 (71%)	11,758 (89%)^b^	1477 (63%)^b^	6139 (69%)	4648 (65%)^b^	
ACE-I/ARB	9630 (62%)	9120 (69%)^b^	1277 (55%)^b^	5458 (61%)	4284 (60%)^b^	
Spironolactone	933 (6%)	1201 (9%)^b^	90 (4%)^b^	546 (6%)	628 (9%)^b^	
Thiazide	3096 (20%)	3173 (24%)^b^	304 (13%)^b^	1797 (20%)	1484 (21%)	
Calcium channel blockers	5354 (35%)	5389 (41%)^b^	622 (27%)^b^	2973 (33%)	2460 (34%)	
Beta blockers	4276 (28%)	4076 (31%)^b^	503 (21%)^b^	2332 (26%)	1961 (27%)	
Clopidogrel	1048 (7%)	988 (8%)	101 (4%)^b^	521 (6%)	531 (7%)	
Digoxin	567 (4%)	370 (3%)^b^	30 (1%)^b^	317 (4%)	335 (5%)	
Acetylsalicylic acid	5472 (35%)	5942 (45%)^b^	645 (28%)^b^	2844 (32%)	2623 (37%)^b^	
Furosemide	2739 (18%)	3162 (24%)^b^	259 (11%)^b^	1458 (16%)	1728 (24%)^b^	

Data is n(%) or mean (SD)

*ACE-I* Angiotensin converting enzyme inhibitor, *ARB* angiotensin receptor blocker, *GLP-1 RA* glucagon like peptide-1 receptor agonists, *SGLT-2* sodium–glucose transporter 2 inhibitor, *DPP-4* dipeptidyl peptidase inhibitor

^a^cardiovascular risk was assed according to EASD/ESC guidelines within the limitation of the registries. DPP-4 is used as reference for all comparisons

^b^Marks values which are significantly different from DPP-4 inhibitors

### Hospitalisation for heart failure

**Fig. 3:**
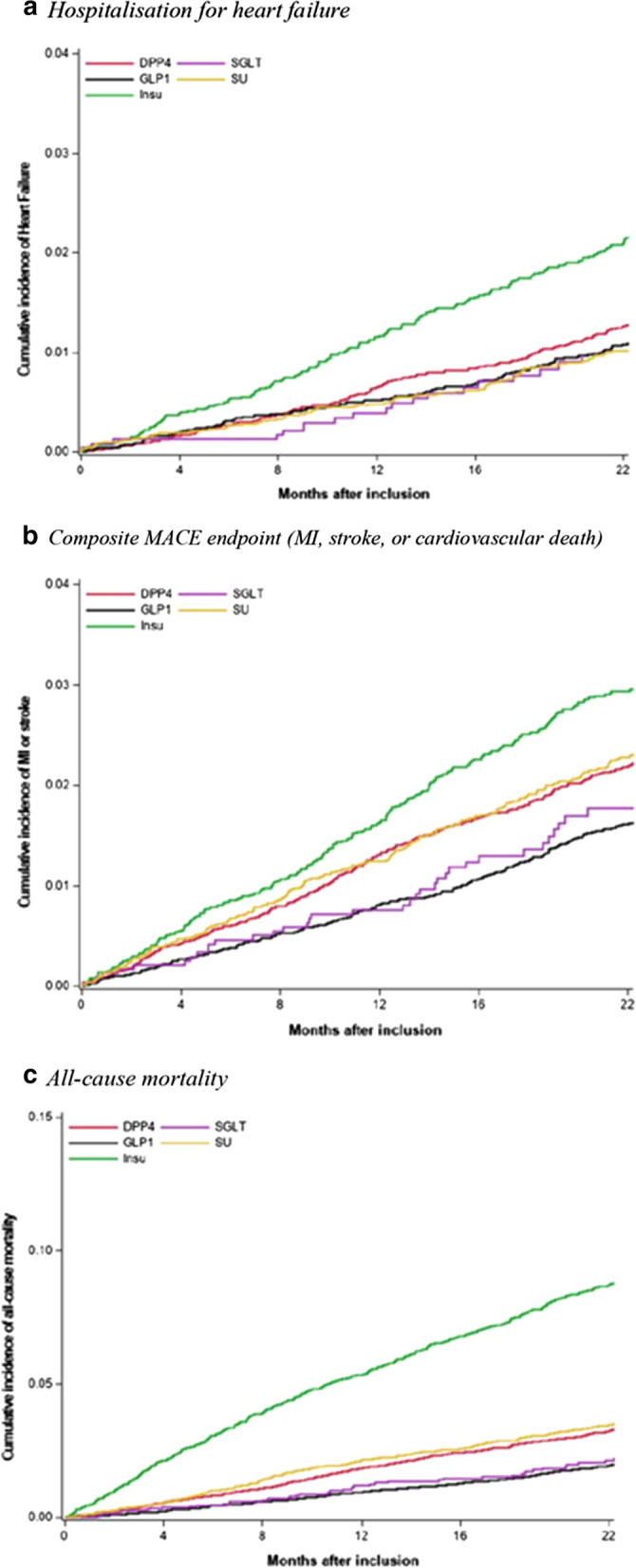
Crude cumulative incidence plot for the events of hospitalisation for heart failure, composite MACE endpoint (myocardial infarction, stroke, or cardiovascular death), and all-cause mortality. *GLP-1* glucagon like peptide-1 receptor agonist, *SGLT* Sodium–glucose transporter 2 inhibitor, *DPP-4* dipeptidyl peptidase 4 inhibitor

**Fig. 4:**
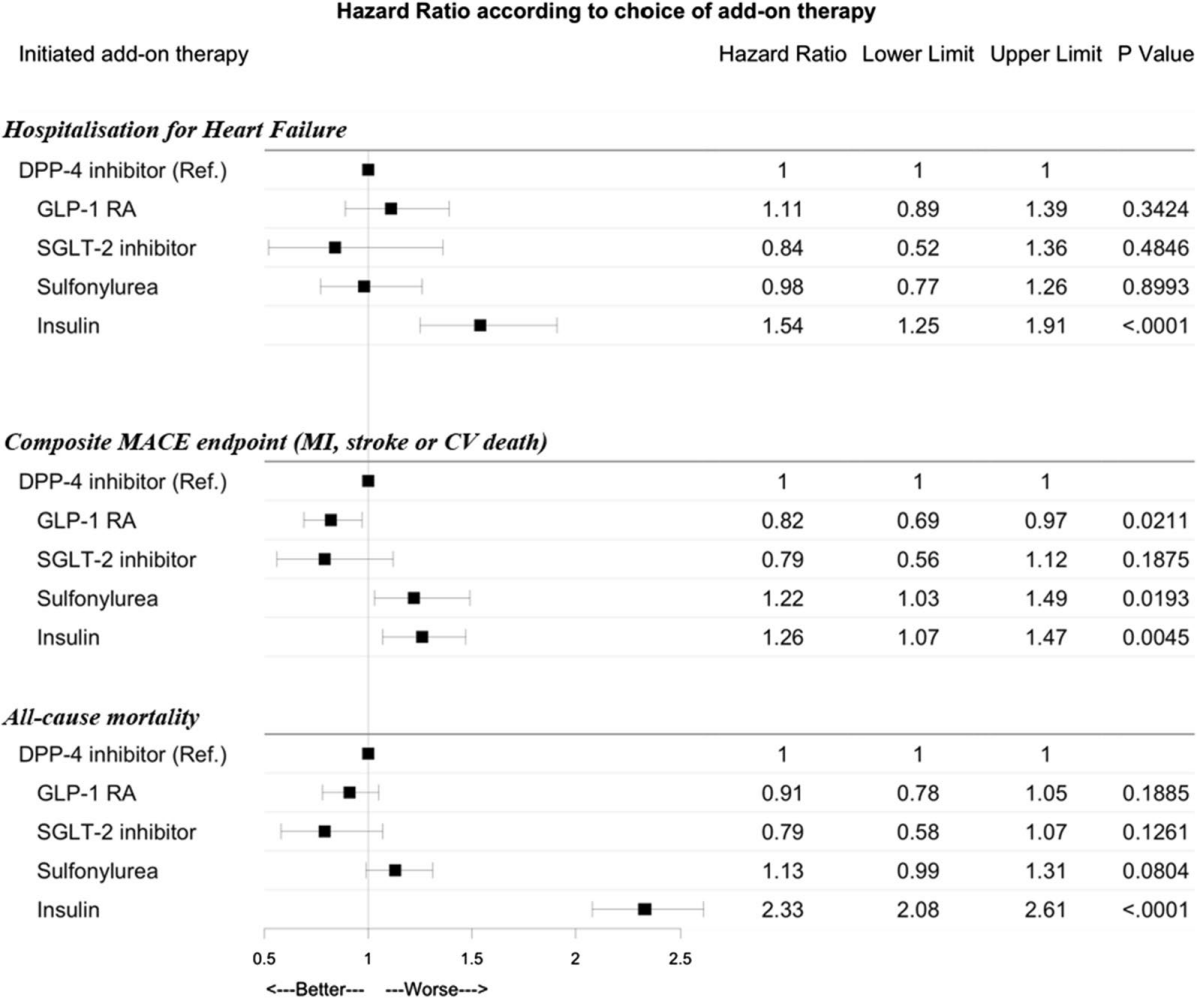
Forrest plot of risk of adverse outcomes according to add-on therapy. Hospitalisation for heart failure, composite MACE endpoint of MI, stroke, or cardiovascular (CV) death, and all-cause mortality according to second-line add-on treatment. *GLP-1 RA* glucagon like peptide-1 receptor agonist, *SGLT-2* sodium–glucose cotransporter 2 inhibitor, *DPP-4* dipeptidyl peptidase inhibitor. The results visualised in the Forest plot is adjusted for age, comorbidities time in metformin monotherapy, year of inclusion, use of statin, CV risk profile, and sex. DPP-4 inhibitors were used as reference

During follow-up, a total of 623 (1.3%) patients were hospitalised for HF. Event rates per 1000 patient years were 5.9 (5.0–6.9) for GLP-1 RA, 4.9 (3.0–7.7) for SGLT-2 inhibitors, 6.9 (6.0–7.9) for DPP-4 inhibitors, 5.5 (4.4–6.7) for SU, and 11.7 (9.9–13.7) for insulin (Table 2). When compared to patients who started add-on DPP-4 inhibitors, initiation of GLP-1 RAs; hazard ratio (HR) 1.11 (95% CI 0.89–1.39), SGLT-2 inhibitors, HR 0.84 (0.52–1.36), and SU, HR 0.98 (0.77–1.26) was not associated with any significant differences in risk of hospitalisation for HF. Insulin was associated with an increased risk of hospitalisation for HF, HR 1.54 (1.25–1.91) (Figs. 3 and 4).

**Table 2:** Event rates of hospitalisation for HF, MACE and all-cause mortality according to second-line therapy

	Heart failure hospitalisation		MACE		All-cause mortality	
	No events	Rate per 1000 py	No events	Rate per 1000 py	No events	Rate per 1000 py
DPP-4 inhibitors	203	6.9 (6.0–7.9)	398	13.5 (12.2–15.0)	542	18.3 (16.8–19.9)
GLP-1 receptor agonists	151	5.9 (5.0–6.9)	261	10.1 (8.9–11.4)	285	11.0 (9.7–12.3)
SGLT-2 inhibitors	19	4.9 (3.0–7.7)	36	9.3 (6.5–12.9)	45	11.6 (8.5–15.5)
Sulfonylurea	94	5.5 (4.4–6.7)	236	13.8 (12.1–15.7)	330	19.2 (17.2–21.4)
Insulin	156	11.7 (9.9–13.7)	265	19.9 (17.5–22.3)	663	49.3 (45.6–53.2)

### MACE (myocardial infarction, stroke or CV death)

The composite endpoint occurred in 1196 (2.5%) patients of which there were 480 myocardial infarctions, 530 strokes, and 257 CV deaths registered for all patients. Event rates per 1000 patient years were 10.1 (8.9–11.4) for GLP-1 RAs, 9.3 (6.5–12.9) for SGLT-2 inhibitors, 13.5 (12.2–15.0) for DPP-4 inhibitors, 13.8 (12.1–15.7) for SU, and 19.9 (17.5–22.3) for insulin (Table 2). GLP-1 RAs were associated with a lower risk of the composite endpoint, HR 0.82 (0.69–0.97), whereas SGLT-2 inhibitors, HR 0.79 (0.56–1.12), SU, HR 1.22 (1.03–1.49) and insulin, HR 1.23 (1.07–1.47) were associated with a similar risk as compared to DPP-4 inhibitors (Figs. 3 and 4).

### All-cause mortality

A total of 1865 (3.9%) patients died, yielding event rates per 1000 patient years of 11.0 (9.7–12.3) for GLP-1 RA, 11.6 (8.5–15.5) for SGLT-2 inhibitors, 18.3 (16.8–19.9) for DPP-4 inhibitors, 19.2 (17.2–21.4) for SU, and 49.3 (45.6–53.2) for insulin (Table 2). Relative to add-on DPP-4 inhibitors, GLP-1 RAs, HR 0.91 (0.78–1.05), SGLT-2 inhibitors, HR 0.79 (0.58–1.07), and SU, HR 1.13 (0.99–1.31) were not associated with significant differences in risk of all-cause mortality. Insulin, HR 2.33 (2.08–2.61) was associated with an increased risk of death (Figs. 3 and 4).

### Changes in treatment regimen during follow-up

At the end of follow up, 82% of patients remained on the glucose-lowering treatment they initiated at study inclusion; 83% for GLP-1 RAs, 80% for SGLT-2 inhibitors, 76% for DPP-4 inhibitors, 82% for SU, and 91% for insulin. Of the patients not initiating insulin therapy, 8% filled at least one prescription for insulin during follow-up; 12% for GLP-1 RAs, 6% for SGLT-2 inhibitors, 11% for DPP-4 inhibitors, and 6% for SU.

### Sensitivity analyses

The overall associated risk of study outcomes did not change considerably for most of the sensitivity analyses (Additional file 1: Table S3–S5). When taking changes in treatment throughout follow-up into account, risk estimates of all-cause mortality were reduced for GLP-1 RA, HR 0.71 (0.57–0.89) and SGLT-2 inhibitors, HR 0.52 0.32–0.87). Conversely, SU was associated with an increased mortality, HR 1.35 (1.13–1.60), Additional file 1: Table S2. Furthermore, in the sensitivity analysis using SU as the reference group as opposed to DPP-4 inhibitors, DPP-4 inhibitors, GLP-1 RAs and SGLT-2 inhibitors were associated with a reduced the risk of the MACE outcome and all-cause mortality (only DPP-4 and SGLT-2 inhibitors). Additional file 1: Table S6).

## Discussion

In this nationwide registry-based cohort study, we examined whether the benefits of GLP-1 RAs and SGLT-2 inhibitors could be extrapolated to patients with T2D at a lower CV risk without prior cardiovascular events who initiated second-line glucose lowering therapy to metformin. We found a low absolute risk of hospitalisation for HF amongst differing second-line treatments, and no significant differences in risk compared to patients who started add-on DPP-4 inhibitors, with the exception of an increased risk with add-on insulin therapy. For the MACE endpoint we found that add-on GLP-1 RA therapy was associated with a ~ 30% lower risk. SU and insulin were associated with a higher risk of MACE. SGLT-2 inhibitors was not associated with a significantly different risk compared to the DPP-4 inhibitor group, but a potential signal towards a benefit of add-on SGLT-2 inhibitors may be argued for, as the number of cases and patients in the SGLT-2 inhibitor group were relatively low. A relatively high proportion of the cohort received GLP-1 RAs as compared to SGLT-2 inhibitors. While speculative, we believe this may be due to the weight-loss effects of the GLP-1 RA as well as the time frame in which the CV outcome trials were presented, and the guidelines were updated. When comparing GLP-1 RAs and SGLT-2 inhibitors to DPP-4 inhibitors, our findings suggest that the benefits of GLP-1 RAs and SGLT-2 inhibitors demonstrated in clinical trials—for patients with T2D and established CV disease, or a high cardiovascular risk profile—may be somewhat lessened in a nationwide population of T2D patients at moderate CV risk. For all-cause mortality, insulin use was associated with a higher risk of death while no differences were observed for GLP-1 RAs, SGLT-2 inhibitors and SUs relative to DPP-4 inhibitors.

The cardiovascular safety of GLP-1 RAs, SGLT-2 inhibitors, and DPP-4 inhibitors has been investigated in RCTs that primarily included patients with established cardiovascular disease or with multiple risk factors [17, 22, 24, 32–34]. The mechanisms explaining the cardiovascular benefits of GLP-1 RAs and SGLT-2 inhibitors are not clear, but they do not seem to be mediated by a reduction in HbA1c [35, 36]. Some studies have suggested that the effects may be driven by individual drugs rather than the class of drugs—both in terms of varying effects demonstrated in clinical trials as well as observational studies [32, 37]. Meta-analyses on SGLT-2 inhibitors, GLP-1 RAs, and DPP-4 inhibitors have not, however, demonstrated significant treatment effect heterogeneity within each drug class [38, 39]. Other observational studies on patients with T2D have for hospitalisation for HF found a similar risk for GLP-1 RAs, a decreased risk for SGLT-2 inhibitors, and an increased risk for DPP-4 inhibitors when compared to a reference group [16, 40–44]. The difference between these results and those found in this study may be a product of the variance in study design, as these studies tend to compare one drug or class of drugs to all other treatment modalities. This simplification may allow specific treatments in the reference group (e.g. insulin) to drive the results. Direct comparisons of SGLT-2 inhibitors and DPP-4 inhibitors have been done in propensity matched analyses by using both Scandinavian and American registries and found SGLT-2 inhibitors to be associated with a reduction in the risk of hospitalisation for HF [25, 42]. The discrepancy between the findings of these and the present study—which did not find SGLT-2 inhibitors to be associated with lower risk of hospitalisation for HF—may be explained by the difference in study design. The aforementioned studies included patient with established cardiovascular disease (30%), and on-going metformin treatment in 60–80% of patients [25, 42]. Additionally, our findings show that the use of SU and insulin were associated with an increased risk of MACE and for insulin all-cause mortality as well. A recent clinical trial has demonstrated cardiovascular non-inferiority when comparing Linagliptin (a DPP-4 inhibitor) with Glimepiride (an SU) [45, 46].

In the sensitivity analysis taking changes to the initial add-on therapy during follow-up into account, GLP-1 RAs and SGLT-2 inhibitors were associated with a reduced risk of all-cause mortality. The discrepancy between the primary analysis and the sensitivity analysis may reflect that changes to the add-on therapy are made when the underlying disease progresses, rather than displaying the direct beneficial results of the treatment. When we excluded patients deemed at high or very high risk, the association between add-on GLP-1 RAs or SGLT-2 inhibitor and CV outcomes were somewhat lessened for hospitalisation for HF and the composite MACE outcome. These analyses may help to explain why our results do not fully mirror the benefits found for SGLT-2 inhibitors and GLP-1 RAs in recent CV outcome trials in patients with established or at high risk of CV disease.

### Limitations

The present study was observational, and the associations observed may not represent causality. The choice of second-line add-on therapy may be influenced by a multitude of factors that cannot fully be captured in the adjusted analyses. The lack of information on important clinical variables including blood glucose and kidney function raises the risk of confounding by indication. Other clinical variables, including BMI, smoking status, and blood pressure were not available. However, surrogate measures for some of these parameters were established and adjusted for based on hospital admissions, e.g. for kidney disease and hypertension. Additionally, the continuous use of metformin indicated that the kidney function was not severely reduced. As comorbidities associated with cardiovascular risk were assessed by ICD-10 coding during hospital admission or outpatient visits, patients with stable vascular disease may have been included and characterised as being at moderate cardiovascular risk. Further, the study may be limited by the follow-up period of 2 years in patients at lower risk. The results of the SGLT-2 inhibitors may be limited by the relatively low number of patients and events in the SGLT-2 inhibitor group. Lastly, We chose not to include glitazones and alpha-glucosidase inhibitors as use of these drugs was low.

## Conclusions

In a nationwide cohort of patients with T2D starting second-line add-on therapy with metformin and with no history of cardiovascular events, initiating either GLP-1 RA or SGLT-2 inhibitor treatment was associated with comparable risks of hospitalisation for HF and death, and a lower risk of the composite endpoint of MI, stroke, or CV death for GLP-1 RAs, relative to DPP-4 inhibitors. Our results suggest that the benefits of these drugs found in CV outcome trials might not be readily extrapolated to patients with T2D at moderate risk of cardiovascular disease. Prospective and pragmatic head-to-head trials comparing cardiovascular risk associated with different glucose-lowering therapies in primary prevention populations are warranted.

## Supplementary information

**Additional file 1: Table S1.** ICD-10 codes used in the study. **Table S2.** A sensitivity analysis, in which patients were followed until a prescription was filled for any anti-diabetic therapy different from the initial treatment. **Table S3.** Follow-up was extended to three years instead of the initial two years in the primary analysis. **Table S4.** Sensitivity analyses: (A) excluding patients a high or very high cardiovascular risk, (B) only including patients at high or very high cardiovascular risk (B). **Table S5.** Sensitivity analyses splitting the cohort in two according to the year of inclusion (A) Patients with a date of inclusion set between 2010 January - 2013 September, (B) Patients with a date of inclusion set between 2013 October - 2017August. **Table S6.** A sensitivity analysis in which sulfonylurea was used as reference as opposed to DPP-4 inhibitors which was used in the primary analysis.

## Data Availability

The data that support the findings of this study are available from Denmark’s Statistics, but restrictions apply to the availability of these data, which were used under license for the current study, and thus are not publicly available. Data are available from the authors upon reasonable request and with permission of Denmark’s Statistics.

## Abbreviations

GLP-1 RAs: Glucagon-like peptide-1 receptor agonists
SGLT-2 inhibitors: Sodium–glucose cotransporter 2 inhibitors
T2D: Type 2 diabetes
DPP-4 inhibitors: Dipeptidyl peptidase-4 inhibitors
SU: Sulfonylurea
HF: Heart failure
MI: Myocardial infarction
ATC: The International Anatomical Therapeutical Chemical System
ICD-10: International Classification of Diseases 10th edition
CV: Cardiovascular
MACE: Major Adverse Cardiovascular Event
