# Supplementary material for: Add-on therapy in metformin-treated patients with type 2 diabetes at moderate cardiovascular risk: a nationwide study
Source: Cardiovasc Diabetol. 2020 Jul 6;19:107. doi: 10.1186/s12933-020-01078-5 (PMC7339487; doi:10.1186/s12933-020-01078-5)
Supplement: Supplementary file 1 — Additional file 1: Table S1. ICD-10 codes used in the study. Table S2. A sensitivity analysis, in which patients were followed until a prescription was filled for any anti-diabetic therapy different from the initial treatment. Table S3. Follow-up was extended to three years instead of the initial two years in the primary analysis. Table S4. Sensitivity analyses: (A) excluding patients a high or very high cardiovascular risk, (B) only including patients at high or very high cardiovascular risk (B). Table S5. Sensitivity analyses splitting the cohort in two according to the year of inclusion (A) Patients with a date of inclusion set between 2010 January - 2013 September, (B) Patients with a date of inclusion set between 2013 October - 2017August. Table S6. A sensitivity analysis in which sulfonylurea was used as reference as opposed to DPP-4 inhibitors which was used in the primary analysis. [file 12933_2020_1078_MOESM1_ESM.docx]

Additional file

|  |  |  |
| --- | --- | --- |
| **Table S1 ICD-10 codes** |  |  |
|  |  |  |
| Atrial fibrillation |  | I48 |
| Cancer |  | C00-C99 |
| Chronic obstructive pulmonary disease |  | J42, J44 |
| Heart failure |  | I11.0, I13.0, I42, I50 |
| Hypertension |  | I10-I15 |
| Ischemic heart disease |  | I20, I25 |
| Microvascular complications |  | E11.2-11.8 |
| Myocardial infarction |  | I21, I22 |
| Peripheral atherosclerosis |  | I70.2-I70.5 |
| Renal disease |  | N03-N08, N11, N14, N18, N19, N25-N29, I12, I13, Q61 |
| Stroke |  | I61, I63, I64 |
|  |  |  |
|  |  |  |
| **ATC codes** |  |  |
| Metformin |  | A10BA02 |
| Insulin |  | A10A |
| GLP-1 RA |  | A10BJ01, A10BJ02, A10BJ03-6, A10BX04 A10BX07, A10BX10, A10BX13 |
| SGLT-2 inhibitor | | A10BD15, A10BD20-21, A10BK01-02, A10BX09, A10BX11-12 |
| DPP-4 inhibitor |  | A10BDO1-08, A10BD10-11, A10BD13 |
| Sulfonylurea |  | A10BB01, A10BB03, A10BB07, A10BB09, A10BB12, A10BX02 |
| Statin |  | C10A |
| ACE inhibitor / Angiontensin receptor blocker |  | C09 |
| Mineralocorticoid receptor antagonist |  | C03D |
| Thiazide |  | C03A |
| Calcium channel blockers |  | C08 |
| Beta blockers |  | C07 |
| Platelet inhibitors |  | B01AC04, B01AC06 |
| Digoxin |  | C01AA05 |
| Loop diuretics |  | C03CA01 |

**Table S2:**

A sensitivity analysis, in which patients were followed until a prescription was filled for any anti-diabetic therapy different from the initial treatment.

| **Follow-up until first change in treatment** | | | |
| --- | --- | --- | --- |
| Treatment | Hospitalisation for HF  HR (95% CI), p-value | MACE (MI, Stroke, CV death)  HR (95% CI), p-value | All-cause mortality  HR (95% CI), p-value |
| DPP-4 inhibitors | 1.00 (Ref). | 1.00 (Ref). | 1.00 (Ref). |
| GLP-1 RA | 1.11 (0.89-1.39), p=0.34 | 0.82 (0.67-0.97), p=0.0210 | 0.71 (0.57-0.89), p=0.003 |
| SGLT-2 inhibitors | 0.84 (0.52-1.36), p=0.48 | 0.79 (0.56-1.12), p=0.19 | 0.52 (0.32-0.87), p=0.013 |
| Sulfonylurea | 0.98 (0.77-1.26), p=0.90 | 1.22 (1.03-1.44), p=0.0193 | 1.35 (1.13-1.60), p<0.001 |
| Insulin | 1.54 (1.25-1.90), p<0.001 | 1.26 (1.07-1.47), p=0.0045 | 3.47 (3.01-4.00), p<0.001 |

**Table S3:**

Follow-up was extended to three years instead of the initial two years in the primary analysis.

| **Three-year follow-up** | | | |
| --- | --- | --- | --- |
| Treatment | Hospitalisation for HF  HR (95% CI), p-value | MACE (MI, Stroke, CV death)  HR (95% CI), p-value | All-cause mortality  HR (95% CI), p-value |
| DPP-4 inhibitors | 1.00 (Ref). | 1.00 (Ref). | 1.00 (Ref). |
| GLP-1 RA | 1.10 (0.92-1.31), p=0.30 | 0.86 (0.74-0.99), p=0.018 | 0.90 (0.80-1.02), p=0.09 |
| SGLT-2 inhibitors | 0.91 (0.60-1.37), p=0.64 | 0.88 (0.65-1.20), p=0.42 | 0.77 (0.59-1.03), p=0.070 |
| Sulfonylurea | 1.06 (0.87-1.30), p=0.58 | 1.21 (1.04-1.39), p=0.0119 | 1.08 (0.96-1.21), p=0.21 |
| Insulin | 1.49 (1.25-1.78), p<0.001 | 1.21 (1.21-1.39), p<0.001 | 2.15 (1.95-2.37), p<0.001 |

**Table S4:**

Sensitivity analyses: (A) excluding patients a high or very high cardiovascular risk, (B) only including patients at high or very high cardiovascular risk (B).

| **A: Excluding patients at high and very high cardiovascular risk** | | | |
| --- | --- | --- | --- |
| Treatment | Hospitalisation for HF  HR (95% CI), p-value | MACE (MI, Stroke, CV death)  HR (95% CI), p-value | All-cause mortality  HR (95% CI), p-value |
| DPP-4 inhibitors | 1.00 (Ref). | 1.00 (Ref). | 1.00 (Ref). |
| GLP-1 RA | 1.41 (0.95-2.11), p=0.09 | 0.70 (0.54-0.96), p=0.0233 | 0.76 (0.79-1.17), p=0.67 |
| SGLT-2 inhibitors | 1.12 (0.53-2.382), p=0.76 | 0.66 (0.35-1.23), p=0.19 | 0.88 (0.59-1.33), p=0.56 |
| Sulfonylurea | 1.02 (0.67-1.54), p=0.93 | 1.05 (0.81-1.62), p=0.73 | 1.25 (1.06-1.47), p=0.0095 |
| Insulin | 1.53 (1.04-2.23), p=0.0296 | 1.27 (1.00-1.63), p=0.053 | 2.7 (2.34-3.12), p<0.001 |
| **B: Only including patients at high and very high cardiovascular risk** | | | |
| DPP-4 inhibitors | 1.00 (Ref). | 1.00 (Ref). | 1.00 (Ref). |
| GLP-1 RA | 0.98 (0.75-1.28), p=0.90 | 0.85 (0.69-1.05), p=0.12 | 0.83 (0.66-1.05), p=0.12 |
| SGLT-2 inhibitors | 0.67 (0.34-1.26), p=0.21 | 0.82 (0.54-1.27), p=0.38 | 0.65 (0.41-1.05), p=0.076 |
| Sulfonylurea | 0.96 (070-1.31), p=0.79 | 1.36 (1.90-1.70), p=0.0064 | 0.88 (0.66-1.16), p=0.36 |
| Insulin | 1.49 (1.15-1.92), p=0.0022 | 1.21 (0.98-1.49), p=0.0749 | 1.73 (1.43-2.10), p<0.001 |

**Table S5:**

Sensitivity analyses splitting the cohort in two according to the year of inclusion (A) Patients with a date of inclusion set between 2010 January - 2013 September, (B) Patients with a date of inclusion set between 2013 October - 2017August.

| **A: First half of follow-up (2010 January - 2013 September)** | | | |
| --- | --- | --- | --- |
| Treatment | Hospitalisation for HF  HR (95% CI), p-value | MACE (MI, Stroke, CV death)  HR (95% CI), p-value | All-cause mortality  HR (95% CI), p-value |
| DPP-4 inhibitors | 1.00 (Ref). | 1.00 (Ref). | 1.00 (Ref). |
| GLP-1 RA | 1.11 (0.89-1.39), p=0.34 | 0.65 (0.55-0.88), p=0.002 | 1.05 (0.86-1.27), p=0.66 |
| SGLT-2 inhibitors | - | - | - |
| Sulfonylurea | 0.84 (0.52-1.36), p=0.48 | 1.11 (0.88-1.39), p=0.39 | 1.15 (0.94-1.40), p=0.18 |
| Insulin | 0.98 (0.76-1.26), p=0.90 | 1.21 (0.98-1.50), p=0.08 | 2.39 (2.01-2.84), p<0.001 |
| **B: Second half of follow-up (2013 October - 2017 August)** | | | |
| DPP-4 inhibitors | 1.00 (Ref). | 1.00 (Ref). | 1.00 (Ref). |
| GLP-1 RA | 1.03 (0.74-1.45), p=0.85 | 0.83 (0.63-1.09), p=0.1762 | 0.91 (0.80-1.03), p=0.12 |
| SGLT-2 inhibitors | 0.85 (0.52-1.39, p=052 | 0.81 (0.56-1.16), p=0.25 | 0.77 (0.58-1.03), p=0.073 |
| Sulfonylurea | 1.02 (0.73-1.43), p=0.91 | 1.36 (1.07-1.73), p=0.012 | 1.13 (1.04-1.28), p=0.043 |
| Insulin | 1.51 (1.14-2.01), p=0.043 | 1.30 (1.03-1.66), p=0.030 | 2.32 (2.10-2.56), p<0.001 |

**Table S6:**

A sensitivity analysis in which sulfonylurea was used as reference as opposed to DPP-4 inhibitors which was used in the primary analysis.

| **Sulfonylurea as reference** | | | |
| --- | --- | --- | --- |
| Treatment | Hospitalisation for HF  HR (95% CI), p-value | MACE (MI, Stroke, CV death)  HR (95% CI), p-value | All-cause mortality  HR (95% CI), p-value |
| Sulfonylurea | 1.00 (Ref). | 1.00 (Ref). | 1.00 (Ref). |
| DPP-4 inhibitors | 1.01 (0.79-1.30), p=0.90 | 0.82 (0.70-0.97), p=0.019 | 0.88 (0.77-1.01), p=0.12 |
| GLP-1 RA | 1.13 (0.86-1.49), p=0.37 | 0.68 (0.56-0.82), p<0.001 | 0.80 (0.68-0.94), p=0.0082 |
| SGLT-2 inhibitors | 0.86 (0.51-1.43), p=0.55 | 0.65 (0.45-0.93), p=0.0205 | 0.69 (0.50-0.96), p=0.043 |
| Insulin | 1.56 (1.21-2.03), p=0.0007 | 1.03 (0.86-1.24), p=0.73 | 2.05 (1.79-2.35), p<0.001 |
